# SOCIALLY ACTIVE OR ISOLATED? OLDER COMMUNITY-DWELLING ITALIANS’ EXPERIENCES DURING THE COVID-19 PANDEMIC

**DOI:** 10.1093/geroni/igad104.2197

**Published:** 2023-12-21

**Authors:** Anne Barrett, Eliana Fattorini, Monica Consolandi

**Affiliations:** Florida State University, Tallahassee, Florida, United States; University of Trento, Trento, Trentino-Alto Adige, Italy; Bruno Kessler Foundation, Trento, Trentino-Alto Adige, Italy

## Abstract

Media coverage of older adults during the COVID-19 pandemic emphasized their experiences of social isolation – a risk that pre-dated the pandemic but was magnified by it. A focus on older adults’ pandemic-induced isolation also is evident in gerontological research. This depiction, however, may not capture the complexity of older adults’ experiences during the pandemic. Our study examined this topic using semi-structured interviews that were conducted with participants in a senior center in a mid-sized northern Italian city in May 2022 (n=23). Our analysis revealed that the pandemic had some negative effects on older adults’ social relationships; however, it also identified strategies that older adults used to maintain and even enhance their connections with others. Participants described reduced in-person interaction with family and friends, stemming from less daily interaction and the cancellation of family gatherings. They compensated for these losses, to some degree, with regular phone and video calls. Participants also described permanently strained relationships with family members and friends, often the result of differing views of COVID-19 vaccines. The pandemic’s challenges, however, enhanced other social relationships through exchanges of moral and practical support. In contrast with the frequent depiction of older adults’ isolation during the COVID-19 pandemic, our data reveal strategies that allowed them to remain socially engaged and active in their communities.

